# GNTF: A Lightweight CNN Robustness Enhancement Method for IoT Devices

**DOI:** 10.3390/s26072207

**Published:** 2026-04-02

**Authors:** Xuan Liu, Benkui Zhang, Jinxiao Wang, Huanyu Bian, Yunping Ge

**Affiliations:** 1 Aerospace Information Research Institute, Chinese Academy of Sciences, Beijing 100094, China; liuxuan@aircas.ac.cn (X.L.); wangjinxiao@aircas.ac.cn (J.W.); bianhy@aircas.ac.cn (H.B.); geyp@aircas.ac.cn (Y.G.); 2 Key Laboratory of Target Cognition and Application Technology (TCAT), Beijing 100190, China; 3 State Key Laboratory of Remote Sensing and Digital Earth, Beijing 100101, China; 4 University of Chinese Academy of Sciences, Beijing 100049, China; 5 School of Electronic, Electrical and Communication Engineering, University of Chinese Academy of Sciences, Beijing 100190, China

**Keywords:** MonoCNN, edge computing, IoT devices

## Abstract

Deploying lightweight convolutional neural networks (CNNs) to provide vision services on resource-constrained Internet of Things (IoT) devices has become the mainstream approach to addressing computing and energy consumption constraints. However, these IoT devices often operate in complex outdoor environments (e.g., fog, rain, and snow), and the quality of the data they collect is easily degraded, causing standard lightweight CNNs to experience a significant performance drop under such corrupted data. To this end, this paper proposes a Generative Nonlinear Transformation Filter (GNTF) method to improve the generalization performance of lightweight CNNs on corrupted data. The core of the GNTF is that only a portion of the filters are used as learnable parameters (named seed filters), while the remaining filters are generated by applying the nonlinear transformation to the seed filters, which is randomly initialized and fixed during training. This design makes the model parameters less dependent on the training data distribution, thereby regularizing the model, mitigating overfitting, and enhancing its robustness to data degradation. The GNTF further analyzes the structural characteristics of lightweight CNNs, showing that significant performance improvements can be achieved simply by replacing the depthwise convolutional modules. Furthermore, this paper examines the properties of various nonlinear transformation functions and finds that model robustness can be improved by applying simple translations. To verify the effectiveness of the GNTF, we conducted extensive experiments on the CIFAR-10/-100, CIFAR-10-C/-100-C, and ICONS-50 datasets, using the MobileNetV2, ShuffleNetV2, EfficientNet, and GhostNet models. The results show that the proposed GNTF can improve the model’s accuracy on corrupted data while reducing the number of trainable parameters in most cases. For example, on the CIFAR-10-C dataset, ShuffleNetV2 with the GNTF improves accuracy by about 3.3% over the original model while slightly reducing the number of trainable parameters.

## 1. Introduction

With the rapid development of Internet of Things (IoT) [1,2] and edge computing [3,4] technologies, deploying lightweight convolutional neural networks (CNNs) [5,6,7,8,9,10,11] on resource-constrained IoT devices (such as smart cameras and sensors) to provide real-time vision services has become a key path to promote the democratization of artificial intelligence. Those IoT devices are widely deployed outdoors, and the data they collect is often affected by complex environmental factors such as fog, rain, and snow, which causes a sharp decline in the performance of standard lightweight CNNs trained on standard data. Therefore, research into enhancing the robustness of CNN models to corrupted data without increasing the computational burden on IoT devices is of paramount importance for ensuring the reliability of edge intelligence services.

Related work, such as that on MonoCNN [12], improves model robustness by fixing a subset of convolutional-layer filters as seed filters and generating the remaining configuration-unlearnable nonlinear transformation function. When generating feature maps, SineFM [13] also applies a nonlinear transformation with nonlearnable hyperparameters to produce additional feature maps from the CNN. SeFA [14] improves the robustness of model fine-tuning by introducing a fine-tuning branch that includes unlearned parameters. However, the above methods still have the following two limitations: (i) they mainly focus on improving the robustness of standard CNNs [15,16,17,18], while research on the robustness of lightweight CNNs containing 1×1 convolutions and depthwise convolutions is still insufficient; (ii) they directly apply the nonlinear transformation function to the filters or feature maps, lacking analysis and comparison of the characteristics of the nonlinear transformation function itself.

To this end, this paper explores a method to enhance robustness in lightweight CNN models. Its core objective is to endow lightweight CNNs with strong generalization capabilities by introducing structured constraints on parameter generation while maintaining, or even reducing, model complexity. To achieve this goal, we propose the Generative Nonlinear Transformation Filter (GNTF) method.

First, we establish the core principle of our method: to break from the traditional practice of learning all filter parameters independently in convolutional layers. We use only a subset of the filters in the network as learnable seed filters, while the parameters of most of the other filters are generated by applying a nonlinear transformation function to these seed filters, which are randomly initialized with hyperparameters and kept fixed during training. This design makes the model parameters less dependent on the statistical distribution of the training data, thereby introducing a data-independent “prior” structure that provides powerful regularization, suppressing overfitting and enhancing stability against out-of-distribution disturbances.

Secondly, we reveal two key prerequisites for the efficient application of the proposed GNTF: the first is the selectivity of layer type: experiments show that applying the generation rule to 3×3 depthwise convolutions responsible for spatial feature extraction in lightweight models yields significant results, while applying it to 1×1 pointwise convolutions yields minimal results. This indicates the specific structural boundaries by which the proposed GNTF is effective. The second is an analysis of the characteristics of nonlinear transformation functions. After comparing various functions, those that pass through the origin yielded the greatest performance improvement. By simply shifting nonlinear transformation functions that do not pass through the origin, the model’s robustness can be improved.

Finally, we validate our findings across several benchmark datasets, including CIFAR-10-C, CIFAR-100-C, and ICONS-50. Experimental results using MobileNetV2, ShuffleNetV2, EfficientNet, and GhostNet as backbone networks show that the GNTF can, following the above principles, simultaneously achieve the goals of reducing burden and increasing efficiency. For example, on the CIFAR-10-C dataset, GNTF-ShuffleNetV2 reduced the number of trainable parameters by about 11% while improving the accuracy by about 3.3%.

In summary, our main contributions are as follows.

- This paper proposes a lightweight CNN robustness enhancement method, the GNTF, that generates filters via fixed nonlinear transformation functions, thereby injecting structured priors independent of the training data and improving robustness without increasing inference overhead.
- This paper finds that, in a lightweight CNN architecture that includes both depthwise and pointwise convolutions, applying the generative nonlinear transformation filter (GNTF) only to the depthwise convolution part most effectively improves the model’s robustness.
- This paper finds that through in-depth analysis of various nonlinear transformation functions, the robustness of the model to data degradation can be further enhanced by simply performing a translation operation.
- The paper demonstrates through experiments that the proposed GNTF, based on the MobileNetV2, ShuffleNetV2, EfficientNet, and GhostNet architectures, achieves high robustness on the distributed datasets CIFAR-10-C, CIFAR-100-C, and ICONS-50.

The rest of the paper is organized as follows. Section 2 reviews the related work. Section 3 describes the proposed method in detail. Section 4 presents our evaluation results, and Section 5 presents the conclusions.

## 2. Related Work

This section introduces lightweight convolutional neural networks (CNNs) and nonlinear transformation techniques for generating model parameters that are most relevant to our paper.

### 2.1. Lightweight CNNs

To adapt to resource-constrained IoT devices, researchers are designing efficient network architectures that replace standard convolutions with more computationally efficient operators. Modular designs based on depthwise separable convolution and its variants have become an industry standard.

For example, Howard et al. proposed MobileNetV1 [5], which introduced depthwise separable convolution into a mainstream CNN. It decomposes standard convolution into two steps, depthwise convolution and pointwise convolution (i.e., 1×1 convolution), which significantly reduces the number of parameters and computational cost. Sandler et al. proposed MobileNetV2 [6], which introduces an inverse residual structure and a linear bottleneck layer: first, 1×1 convolutions are used to expand the number of channels, then depthwise convolutions are used for spatial filtering, and finally 1×1 convolutions are used to compress the number of channels, while linear activation is used to avoid loss of nonlinear information. MobileNetV3 [19] further optimizes the balance between accuracy and speed by combining neural architecture search with the new activation function. Zhang et al. [7] proposed ShuffleNetV1, which addresses the computational overhead of 1×1 pointwise convolutions by using grouped pointwise convolutions and by innovatively introducing channel shuffling to promote information flow between groups and ensure accuracy. ShuffleNetV2 [8], starting from the actual hardware efficiency, such as memory access cost, proposes four practical design principles to build a more efficient lightweight model. Tan et al. proposed EfficientNet [9,10], which systematically balances depth, width, and resolution through a composite scaling method. Its basic building block, MBConv, is based on the inverse residual structure of MobileNetV2. Han et al. proposed GhostNet [11], offering a novel perspective by arguing that feature maps contain substantial redundancy. It generates a portion of the “ghost” feature map via inexpensive linear operations (e.g., depthwise convolution), then concatenates it with the feature map produced by the original convolution, yielding a sufficient feature map with fewer parameters and achieving a lightweight design.

The common paradigm in these works is to use 1×1 convolutions for channel-level information fusion and dimensionality transformation and to combine them with depth-separable 3×3 convolutions to efficiently extract spatial features, thereby achieving a significant reduction in parameters and floating-point operations while maintaining competitive model performance. However, its optimization objectives primarily focus on achieving high accuracy on clean, standardized benchmark datasets, with relatively little consideration for the model’s robustness in the complex physical world.

However, lightweight CNN models are widely deployed on outdoor IoT devices that typically process environmentally sensitive data. Standard lightweight CNN models perform poorly when handling such data. Therefore, this paper aims to reduce overfitting by fixing a portion of the model parameters so they are not updated during training and to improve the model’s robustness by generating a regularized model via parameter settings.

### 2.2. Nonlinear Transformation Techniques for Generating Model Parameters

Recent research has explored methods to enhance model robustness using seed filters, aiming to improve the robustness and efficiency of convolutional neural networks, and has achieved promising results. For example, MonoCNN [12] reduces over-reliance on training data by fixing a portion of the filters in the convolutional layer as seed filters, while the remaining filters are generated from the seed filters through nonlearnable nonlinear transformations. It enhances the CNN model’s robustness by introducing regularization via transformation rules. SineFM [13] first generates the seed feature maps based on the seed filters and then derives other feature maps through the seed feature maps and a nonlinear transformation, thereby improving robustness while reducing computational overhead. SeFA [14] is designed for model fine-tuning scenarios and features a fine-tuning branch based on seed filters and non-learnable transformations, which effectively improves the stability and performance of fine-tuning.

However, the above methods are designed for conventional convolutional structures and do not fully account for the characteristics of the widely used 1×1 and depthwise 3×3 convolutions in lightweight CNNs. Their enhancement mechanisms are difficult to directly transfer to such networks. In addition, they apply the nonlinear transformation function directly to the filters or feature maps, without analyzing or comparing their statistics. Therefore, this paper aims to study model augmentation methods for lightweight CNNs to adapt to their structural characteristics and improve performance and efficiency.

In addition, many researchers have proposed effective methods to enhance the robustness of CNN models, providing important inspiration for this paper. For example, Momeny et al. [20] enhanced the model’s robustness to input perturbations by introducing a noise-mapping layer and an adaptive resizing layer into the CNN. CondConv, proposed by Yang et al. [21], improves the robustness and expressive power of the model while maintaining efficient inference by learning a specialized convolutional kernel for each sample. Gong et al. [22] introduced a random feature concealment mechanism into the shallow layers of CNNs, which effectively improved the model’s robustness to adversarial examples. Chakraborty et al. [23] proposed a fully complex residual CNN that directly processes complex data in the frequency domain, thereby improving the model’s robustness to frequency-domain perturbations. The HybridAugment++ proposed by Yucel et al. [24] significantly improves the model’s robustness to common corruptions by reducing the CNN’s reliance on the image amplitude component and enhancing its use of phase information. These diverse and inspiring works have collectively spurred our in-depth analysis of the relationship between the mathematical properties of nonlinear transformation functions and model robustness.

## 3. The Proposed Method: GNTF

### 3.1. Motivation

Our motivation mainly comes from three questions: (i) In the era of Internet of Everything, lightweight CNNs will be widely deployed on outdoor IoT devices due to their low resource consumption advantage. How can we make the lightweight CNN robust to environmental data-processing effects? (ii) Existing methods, such as MonoCNN, improve the robustness of standard CNN models by generating filters using the nonlinear transformation function. Could the idea behind MonoCNN be used to enhance the robustness of lightweight CNN models? (iii) In the process of generating filters through nonlinear transformations, what characteristics of nonlinear transformation functions can bring optimal performance?

In a MonoCNN-based convolution layer, a small set of trainable seed filters $W_{s}\in R^{c_{s}\times c_{i}\times k\times k}$ is first defined, where $c_{s}$ and $c_{i}$ denote the number of seed filters and input channels, respectively, and *k* denote the filter size. Nonlinear transformation functions f(·) are then applied to the seed filters to generate a large number of non-trainable filters $W_{g}\in R^{c_{(r-1)\times s}\times c_{i}\times k\times k}$. This process can be expressed as:

(1) $$\begin{array}{cc} W_{g} & =\left[f_{1}\left(W_{s}\right),\ldots ,f_{r-1}\left(W_{s}\right)\right]\\ \; & =[W_{g_{1}},\ldots ,W_{g_{r-1}}] \end{array}$$

where $r=\frac{c_{o}}{c_{s}}$ is the filter reduction ratio, and all functions share the same form but have different hyperparameters.

The seed filters and the generated filters are then concatenated to form the full filter set $W\in R^{c_{o}\times c_{i}\times k\times k}$. This process can be expressed as:

(2) $$\begin{array}{c} W=\left[W_{s},W_{g}\right] \end{array}$$

Finally, standard convolution is applied to the input *x* using the complete filter set, producing the output feature map *y*:

(3) $$\begin{array}{c} y=W*x \end{array}$$

MonoCNN reduces the number of trainable parameters by randomly initializing the hyperparameters of the nonlinear transformation function and keeping them fixed throughout training. The fixed nonlinear transformation functions also provide implicit regularization, thereby improving robustness. However, MonoCNN is designed for standard CNNs such as ResNet [16] and VGG [15], yet it still relies on computationally costly 3×3 standard convolutions during inference. As a result, it does not reduce the inference cost when deployed on resource-limited IoT devices.

### 3.2. Design of GNTF

To accommodate resource-constrained IoT environments, lightweight CNN models such as MobileNets [5,6], ShuffleNets [7,8], EfficientNet [9], and GhostNet [11] have been developed, replacing standard convolutions with depthwise separable convolutions. A depthwise separable convolution first performs a depthwise convolution, in which each 3×3 filter is applied to a single input channel, followed by a pointwise convolution using 1×1 filters to fuse channel information. This design significantly reduces the number of parameters and computation while maintaining competitive accuracy, making it widely used in IoT scenarios. IoT environments are highly variable, and the collected images often contain noise and blur. Depthwise separable architectures suffer notable performance degradation under such corruptions. Given that MonoCNN and SineFM use nonlinear transformation functions to improve model robustness, we now address the first question of whether to use fixed hyperparameters to generate filters that enhance robustness.

Based on the foregoing discussion, the second question naturally arises: Can the filter generation method in MonoCNN be directly transferred to lightweight CNNs? In depthwise separable convolutional structures, 1×1 convolutions primarily adjust feature dimensions and fuse cross-channel information, while 3×3 depthwise convolutions focus on extracting spatial features. Since the hyperparameters of the nonlinear transformation functions remain fixed after random initialization, their behavior is more focused on extracting spatial structural features with generalizability. Therefore, we believe that the GNTF is more suitable for 3×3 depthwise convolutions responsible for spatial feature modeling and not suitable for 1×1 convolutions whose main function is to flexibly integrate channel information.

To verify this hypothesis, we designed experiments using GNTF-MobileNetV2 and GNTF-ShuffleNetV2. Under fixed hyperparameter settings, we systematically evaluated the performance of different methods on CIFAR-10 and its corrupted version, CIFAR-10-C.

As shown in Table 1, we evaluated the combined effects of applying the GNTF to different modules in lightweight CNNs. The experimental results conform to our previous analysis: since 1×1 convolution and 3×3 depthwise convolution perform completely different functions in the network—the former is responsible for cross-channel information fusion and projection, while the latter focuses on the extraction of spatial features—the parameter generation strategy has a significantly different impact on them. Specifically, the model performs best on the robust CIFAR-10-C benchmark when only standard 3×3 depthwise convolutions are replaced with our generative architecture. This configuration achieved the highest performance of all replacement combinations on GNTF-MobileNetV2. The same conclusion was strongly validated on the GNTF-ShuffleNetV2 model, which achieved an accuracy of 74.82% in this setting.

In comparison, replacing the 1×1 convolution module or both modules simultaneously does not improve performance as much as replacing only the 3×3 convolution module. This fully demonstrates that the advantages of our proposed filter-generation method based on nonlinear transformation are primarily reflected in the feature-extraction stage. This method effectively introduces beneficial constraints or inductive biases into spatial feature learning without compromising necessary channel interactions, thereby significantly enhancing the model’s robustness to corrupted inputs. Note that this conclusion provides clear, practical guidance for designing efficient parameterization methods for lightweight CNNs.

**Figure 1:**
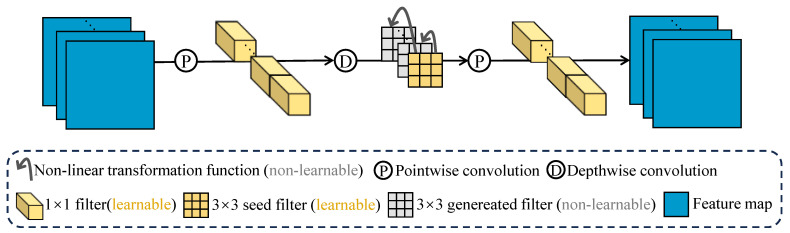
GNTF-based module of lightweight CNN.

Figure 1 illustrates how the GNTF is applied within a lightweight module based on depthwise convolution. In architectures such as ShuffleNetV2 and MobileNetV2, a depthwise-separable-convolution module typically consists of a 1×1 pointwise convolution, a 3×3 depthwise convolution, and another 1×1 pointwise convolution. The 1×1 convolutions mainly adjust feature dimensions and fuse cross-channel information, whereas the 3×3 depthwise convolution is responsible for spatial feature extraction. Therefore, to improve the model’s ability to extract features from noisy images, we apply the nonlinear transformation only to the 3×3 depthwise convolution to generate a large number of filters. The details of our method are described as follows.

First, a 1×1 pointwise convolution is applied to the input *x*, producing the output of the first layer y′. This process is written as:

(4) $$\begin{array}{c} y\prime =W_{1}^{pw}*x \end{array}$$

where pw indicates the pointwise convolution operation.

Second, in the 3×3 depthwise convolution layer of the module, we specify $\frac{c_{o}}{r}$ learnable seed filters $W_{s}\in R^{c_{s}\times c_{i}\times k\times k}$ where $c_{i}$, $c_{o}$, and $c_{s}$ denote the numbers of input channels, output channels, and seed filters, respectively, and $r=\frac{c_{o}}{c_{s}}$ is the reduction ratio of learnable filters. The two pointwise convolution layers remain unchanged. We then use r−1 nonlinear transformation functions f(·) to generate r−1 groups of non-learnable filters from the seed filters. These transformed filters are concatenated to form $W_{g}\in R^{c_{g}\times c_{i}\times k\times k}$. All the functions share the same functional form but differ in their hyperparameters, which are randomly initialized. The seed filters and the generated filters are concatenated to obtain the full set of 3×3 depthwise filters $W^{dw}\in R^{c_{o}\times c_{i}\times k\times k}$. Applying these filters to the intermediate output y′ yields the depthwise-convolution output y″:

(5) $$\begin{array}{cc} y\prime \prime & ={\left[W_{s},\left[f_{1}\left(W_{s}\right),f_{2}\left(W_{s}\right),\ldots ,f_{r-1}\left(W_{s}\right)\right]\right]}^{dw}*y\prime \\ \; & ={\left[W_{s},W_{g}\right]}^{dw}*y\prime \\ \; & =W^{dw}*y\prime \end{array}$$

where dw indicates the depthwise convolution operation.

Finally, a 1×1 pointwise convolution is applied to y″ to produce the final module output *y*:

(6) $$\begin{array}{c} y=W_{2}^{pw}*y\prime \prime \end{array}$$

Our method leverages nonlinear transformation functions with different hyperparameters to generate non-learnable filters. During training, only the seed filters and the 1×1 filters are updated. Therefore, our approach enhances model robustness while further reducing the number of learnable parameters.

After determining that using nonlinear transformation functions to generate filters can improve model robustness and that the MonoCNN mechanism is not suitable for direct porting to lightweight CNNs, we face a third key question: Which characteristics of the nonlinear transformation function yield the best performance when generating filters?

To explore this issue, we first note that the monomial function used in MonoCNN passes through the origin and is symmetric about it. In our generative framework—that is, based on a set of seed filters, the remaining filters are generated through a nonlinear transformation function with fixed hyperparameters—we infer that the core advantage of making the nonlinear transformation function pass through the origin (or return the phase to zero) is that it ensures the zero bias and symmetry of the generation process, thereby improving the overall quality of the final generated filter as a feature extractor.

Passing through the origin ensures the purity of the generated baseline. When the seed filter’s encoded input is zero, the transform output is also zero. This eliminates the possibility that the generation mechanism introduces a constant background filter or a systematic offset out of thin air, making the generated filter family entirely driven by meaningful variations in the seed filter. Thus, the generating function has zero mean and responds exclusively to structural changes in the image (e.g., edges and textures) rather than to uniform-brightness regions, thereby significantly improving the signal-to-noise ratio of the feature representation.

Symmetry, on the other hand, endows the generation process with clear and controllable mathematical behavior. Fixed and symmetric about the origin, nonlinear functions (e.g., odd functions) have well-defined output ranges and transformation modes. This makes the generation process from the seed filters to the entire filter family structured and predictable, allowing designers to precisely control the spectral coverage and geometric shape of the generated filter family based on the function characteristics and to build a stable, complementary, and highly interpretable set of fixed feature extractors without training. This feature is particularly important for deployment scenarios that require high reliability and low computational overhead.

**Figure 2:**
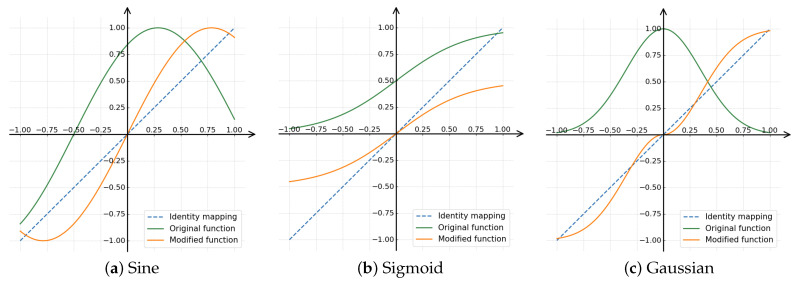
Visualization of different NLFs. (**a**) Sin function: f(x) = sin(βx+ϕ) to f(x) = sin(βx). (**b**) Sigmoid function: f(x) = $\frac{1}{1+e^{-\beta x}}$ to f(x) = $\frac{1}{1+e^{-\beta x}}-0.5$. (**c**) Gaussian function: f(x) = $e^{{-(\beta x)}^{2}}$ to f(x) = $-sign\left(x\right)\left(e^{{-(\beta x)}^{2}}-1\right)$.

Based on the above analysis, in order to verify the importance of zero bias and symmetry, we specifically set the phase of the Sine function to 0 and made a simple translation of the Sigmoid and Gaussian functions so that they pass through the origin and are symmetrical about the origin (the visualization comparison of the functions before and after the correction is shown in Figure 2). Subsequently, we evaluated the effectiveness of using these modified functions on four lightweight models using the CIFAR-10-C dataset.

The experimental results are shown in Table 2. The analysis shows that the Mono function, which possesses the aforementioned characteristics, already achieves good results. By modifying the Sine, Sigmoid, and Gaussian functions to satisfy specific conditions, we improved the model’s robustness and accuracy. For example, on the ShuffleNetV2 model, the modified Gaussian function improved the accuracy from 74.74% to 76.82%; On the GhostNet model, the accuracy of the modified Gaussian function improved from 73.11% to 74.50%, even surpassing the Mono function’s results. These consistent performance improvements demonstrate that ensuring the transform function has zero bias, passes through the origin, and is symmetric about it is a key, necessary condition for improved performance when generating filters based on nonlinear transforms.

Thus far, we have systematically discussed and answered the three key questions initially raised in this article: (i) Using a nonlinear transformation with fixed hyperparameters and no updates to generate filters can effectively enhance the robustness of the model; (ii) The MonoCNN method is not suitable for direct porting to the lightweight CNN. Instead, simply replacing the depthwise convolution module can improve robustness while maintaining efficiency. (iii) A suitable nonlinear transformation function should have the core characteristics of passing through the origin and being symmetric about the origin.

### 3.3. Forward Propagation Procedure of GNTF-Based Module

The forward propagation procedure is presented in Algorithm 1. First, a 1×1 pointwise convolution is applied to the input *x* to generate the output of the first layer y′. Second, r−1 nonlinear transformation functions f(·) are applied to $\frac{c_{o}}{r}$ learnable seed filters $W_{s}$ to generate r−1 groups of non-learnable filters in the 3×3 depthwise convolution layer of the module. These generated filters are concatenated to form $W_{g}$. Next, the seed filters and the generated filters are concatenated to obtain the full set of 3×3 depthwise filters $W^{dw}$. Applying these filters to the intermediate output y′ yields the depthwise-convolution output y″. Finally, a 1×1 pointwise convolution is applied to y″ to produce the final module output *y*.
**Algorithm 1** Forward propagation of GNTF-based Module    **Input:** The GNTF-based lightweight Module M, in_channels $c_{i}$, out_channels $c_{o}$, reduction ratio *r*, range of non-linear mapping hyperparameters *a* and *b*, output of last layer x.**Output:**   The output y of GNTF-based lightweight module**Initialization**:Generate r−1 hyperparameters of non-linear mappings based on *a* and *b***Forward Propagation**:Compute $y\prime =W_{1}^{pw}*x$Generate the remaining filters $W_{g}=f\left(W_{s}\right)$Concatenate $W_{s}$ and $W_{g}$ into $W^{dw}$;Compute $y^{\prime \prime }=W^{dw}*y\prime$Compute $y=W_{2}^{pw}*y\prime \prime$**Return** y;

### 3.4. Discussion

GNTF differs from MonoCNN in the following three aspects:

First, unlike MonoCNN, which applies filters to standard convolutions, GNTF is motivated by a structure analysis of lightweight CNNs. Through experiments, we found and demonstrated (Table 1) that the effectiveness of generative constraints is architecture-dependent: applying the generative mechanism only to the 3×3 depthwise convolution responsible for spatial feature extraction improves robustness, whereas applying it to the 1×1 pointwise convolution has little effect. This discovery reveals a robust “sweet spot” unique to lightweight networks, an insight that the MonoCNN cannot provide.

Second, GNTF conducted a more in-depth analysis of the nonlinear transformation function itself. MonoCNN directly uses existing functions without analysis, whereas we systematically studied the mathematical properties of these functions (Figure 2 and Table 2) and identified the key condition for achieving optimal performance: functions that pass through the origin and are symmetric about it can enhance robustness. By simply shifting a non-zero center function (such as Sigmoid or Gaussian), we achieved performance that even surpassed the Monomial function used by MonoCNN. This is not a simple porting effort but a methodological advancement that is universally applicable across architectures.

Third, the research objectives are fundamentally different: MonoCNN focuses on reducing the transmission overhead between the cloud and the device, whereas GNTF aims to inject structured priors into local IoT models to regularize them and mitigate unknown environmental degradation, thereby achieving parameter reduction and efficiency improvements across multiple lightweight models.

## 4. Experiments

This section presents the experimental setup, including the datasets, baselines, and implementation details. We then provide comparative evaluations followed by detailed analyses.

### 4.1. Experimental Setup

**Datasets.** Four benchmark datasets are used to evaluate our proposed GNTF: CIFAR-10, CIFAR-10-C, CIFAR-100-C, and Icons-50. CIFAR-10-C, CIFAR-100-C, and Icons-50 are used to evaluate robustness under impaired conditions, while CIFAR-10 is used as the standard classification dataset.

**Figure 3:**
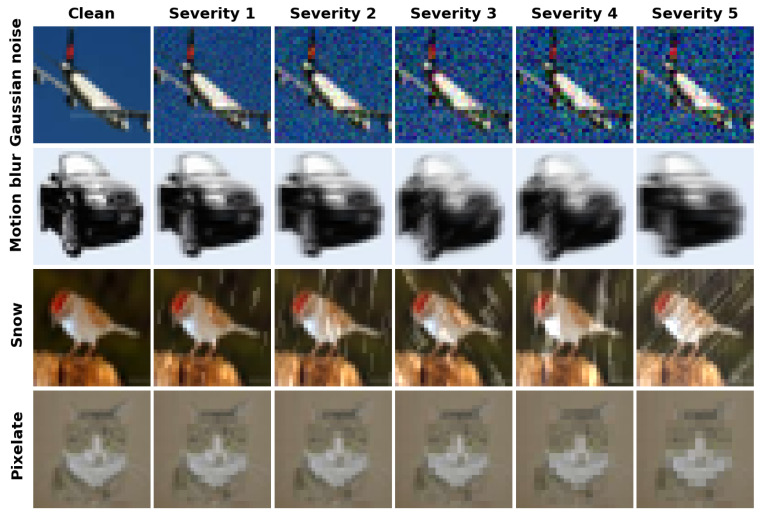
Visualization examples of commonly observed corruptions.

- CIFAR-10 [25] is a widely used multi-class dataset of natural images for image classification. Each dataset contains 50,000 training images and 10,000 test images with 10 and 100 categories, respectively. All images have a resolution of 32×32 pixels.
- CIFAR-10-C and CIFAR-100-C [26] are robustness benchmark datasets used for robustness evaluation. They are constructed by applying synthetic damage and perturbations to the CIFAR-10 and CIFAR-100 test sets. These images were affected by 19 types of damage, covering four main categories: noise, blue, weather, and digital. Each damage type had five severity levels, with 10,000 images per level, for a total of 50,000 images per type. The total number of damaged images across all 19 types reached 950,000, each measuring 32×32 pixels. See Figure 3 for visualization.
- Icons-50 [26] is an icon-style robustness dataset comprising 50 categories and 10,000 images, originally at 120×120 resolution, collected from multiple companies. All images are resized to 32×32 pixels. Following prior work, icons from one of the companies (Apple, Facebook, Google, or Samsung) are used as the test set, while icons from the remaining companies constitute the training set.

**Baselines.** We select four widely used lightweight architectures, MobileNetV2 [6] ShuffleNetV2 [8], EfficientNet [9], and GhostNet [11] as benchmarks. Our method is built upon these architectures and is directly compared against them.

**Implementation Details.** We implement our method using Python 3.10 and PyTorch 2.7.1 with CUDA 11.8 and conduct all experiments on NVIDIA RTX 4090 GPUs. To accommodate 32×32 input images, we adjust the stride of the first standard convolution and the second depthwise convolution in MobileNetV2, as well as the stride of the first convolution in ShuffleNetV2, from 2 to 1. In addition, the output head is modified accordingly for each dataset. We set the filter reduction ratio *r* to 2, 4, 8, 16 and search over the hyperparameter range of the nonlinear transformation functions, with β varying from 0 to 7, using a grid search. We use SGD as the optimizer for all experiments, with a weight decay of $5\times 10^{-4}$. A cosine-annealed learning rate schedule is used, and the initial learning rates for MobileNetV2 and ShuffleNetV2 are set to 0.05 and 0.01, respectively.

We deploy a prototype system on a resource-constrained Raspberry Pi 4B, equipped with a Broadcom BCM2711 quad-core ARM Cortex-A72 CPU (1.50 GHz) and 8 GB of memory. To accurately measure energy consumption during model inference, the system uses a USB-C power meter, POWER-Z KM003C, to record power and energy consumption data in real time. The device-side inference backend is implemented using ONNX Runtime. Note that multiply–accumulate operations (MACs) are measured in PyTorch-CPU.

### 4.2. Experimental Results

#### 4.2.1. Results on Corrupted Datasets

Table 3 shows the performance comparison of each method on the corrupted datasets CIFAR-10-C, CIFAR-100-C, and ICONS-50, based on MobileNetV2, ShuffleNetV2, EfficientNet, and GhostNet models.

(i) The GNTF method has shown advantages on a variety of lightweight models and cross-domain robustness benchmarks. The GNTF achieves improved accuracy across all model-dataset combinations with almost no increase or even a decrease in the number of trainable parameters. For example, with ShuffleNetV2, a reduction of approximately 0.29M trainable parameters achieved accuracy gains of +3.34%, +0.95%, and +0.23% on CIFAR-10-C, CIFAR-100-C, and ICONs-50, respectively. This indicates that the method improves the quality and robustness of feature representations by introducing a nonlinear transformation function with origin symmetry, thereby injecting effective inductive bias and regularization constraints into the generation filter.

(ii) The most improvement was achieved on CIFAR-10-C: all models achieved substantial improvements on this dataset (+2.14%∼+3.34%). This dataset contains various artificially synthesized erosion types (noise, blur, weather changes, etc.), demonstrating that the filters generated by the GNTF can effectively enhance the model’s resistance to low-level visual interference. This is closely related to the function’s frequency-awareness and local-structure-preservation characteristics. The improvement on CIFAR-100-C is moderate, with gains of +0.19% to +0.95%. The dataset has more granular categories and higher semantic complexity, indicating that the method has also improved the robustness of mid- to high-level semantics to some extent, but the room for improvement is constrained by the task’s complexity. The improvement on ICONs-50 is relatively small, with gains of +0.06% to +0.16%. The dataset is relatively small, and there are significant differences in style domain between the training set (icons) and the test set (real-world scene icons). This indicates that the current method primarily enhances the model’s robustness to content changes by fixing hyperparameters and imposing symmetry constraints, while there remains room to improve its generalization across domains such as style and texture. This further confirms that the method is better suited to content-driven robustness enhancement.

(iii) ShuffleNetV2 and GhostNet benefited the most, especially on CIFAR-10-C. This may be related to its channel recombination and feature-reuse mechanisms. The structured filters generated by the GNTF can work more effectively with these lightweight operations and enhance feature diversity. EfficientNet and MobileNetV2 both achieved consistent improvements, indicating that the method is well-suited to a range of lightweight architectures and is not limited to specific module designs.

In summary, the GNTF improves the robustness of lightweight CNNs in various damage types and cross-domain scenarios without increasing computational burden by introducing a nonlinear transformation function with zero bias and symmetry.

#### 4.2.2. Results on Data of Different Corruption Types and Levels

Table 4 compares the robustness of the standard method and the GNTF method on the EfficientNet architecture with fine-grained performance, covering 19 types of damage, each with a severity level of 1 to 5. Overall, GNTF-EfficientNet achieves an average accuracy of 73.45%, which is 3.19 percentage points higher than the standard EfficientNet’s 70.26%, further validating the effectiveness of the proposed method. At higher levels of damage (severity levels 4 and 5), the GNTF demonstrates a more significant advantage. At severity level 5 (the most severe damage), the average accuracy of GNTF-EfficientNet is 56.76%, significantly higher than the standard model’s 51.91%, representing an improvement of 4.85 percentage points. It performs particularly well in mitigating noise-related degradation: for example, the accuracy improved from 16.91% to 27.68% for Gaussian noise, from 21.33% to 32.70% for impulse noise, and from 21.39% to 34.37% for shot noise. This demonstrates that the method, using a filter generated by a nonlinear function with zero bias and symmetry, can more effectively suppress severe noise interference and enhance the stability of feature representations.

In terms of the types of disturbances, the GNTF shows consistent improvement across a variety of perturbations. In addition to noise-related disturbances, the GNTF generally outperforms the baseline across blurring types (e.g., defocus and motion blur), natural disturbances (e.g., fog and frost), and compression distortions (e.g., JPEG compression). This further confirms that the introduced inductive bias—a nonlinear transformation that passes through the origin and is symmetric—can guide the generation of more generalizable filters, thereby maintaining stronger feature-extraction capabilities under diverse image corruptions.

It is worth noting that there was no performance improvement under a few additional global disruptions (e.g., brightness adjustment and pixelation). Our explanation is as follows: The GNTF generates filters with zero-mean characteristics and local-structure sensitivity via zero-crossing and symmetry constraints. This makes the model more robust to changes in high-frequency information such as local texture and edges (e.g., noise, blur, weather interference). However, global perturbations, such as brightness adjustments, change the image’s average pixel value, primarily affecting its global statistical distribution. The zero-mean filter generated by the GNTF is inherently insensitive to absolute brightness levels—meaning that when brightness changes occur as disturbances, the filter cannot detect them through its response and therefore cannot provide targeted protection. Perturbations such as pixelation are characterized by regular structural distortions (e.g., jagged edges and block artifacts caused by undersampling) rather than random noise. The GNTF generator filter is based on a linear combination of seed filters. Its diversity is limited by the number of seed filters, which may be insufficient to cover the feature space of such regular distortions, thereby reducing its protective effect.

In summary, the GNTF not only outperforms standard methods in overall robustness but also shows greater robustness when the degree of damage intensifies, which is closely related to its nonlinear transformation mechanism based on fixed hyperparameters. This mechanism avoids overreliance on training data and provides regularization for the model through the function’s mathematical constraints, thereby maintaining good performance even under severe, unseen disturbances. This conclusion is consistent with the earlier view that passing through the origin and symmetry can improve feature purity, further illustrating the important role of these functional characteristics in enhancing the model’s robustness to interference. In addition, based on the above analysis, we believe that GNTF is particularly well-suited to scenarios where local structural degradation is the primary issue, such as image enhancement for outdoor surveillance cameras affected by rain and snow.

#### 4.2.3. Resource Evaluation in On-Device Inference

The experimental results in Table 5 confirm the effectiveness of the GNTF: it preserves the original inference efficiency while improving the model’s robustness. The reason is that the GNTF generates additional filters from seed filters via nonlinear transformations, rather than altering the inherent structure of classic lightweight CNNs. This plug-and-play feature ensures that performance improvements do not introduce additional inference latency or computational burden.

### 4.3. Ablation Study

#### 4.3.1. Model Training Convergence

**Figure 4:**
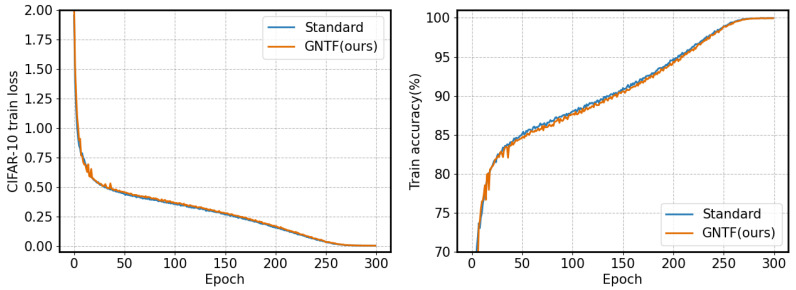
Convergence and accuracy during training on cifar-10 dataset based on the EfficientNet.

**Figure 5:**
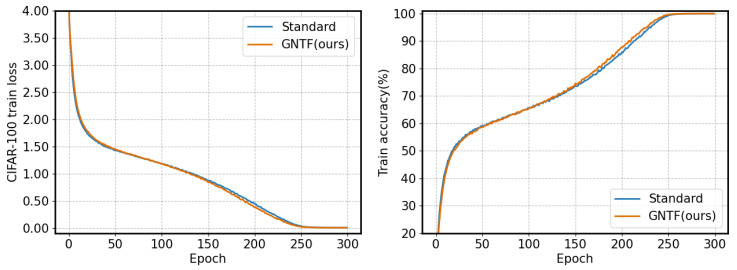
Convergence and accuracy during training on cifar-100 dataset based on the EfficientNet.

To verify the impact of the proposed method on model convergence speed, we present training results and accuracy on CIFAR-10 and CIFAR-100 using the EfficientNet model, as shown in Figure 4 and Figure 5, respectively.

As shown, the model based on the GNTF method and the standard model exhibit highly consistent convergence dynamics during training. Specifically, on the CIFAR-100 dataset (second table), the training loss and accuracy of the two methods are almost identical across all checkpoints from the start of training (Epoch 0) to the end (Epoch 300). This high degree of synchronization across stages clearly indicates that the GNTF method has not altered the model’s overall convergence trajectory or speed. Therefore, the proposed GNTF method—which generates filters through nonlinear transformations of fixed hyperparameters—significantly improves model robustness (as shown in the tables above) without introducing additional training difficulty or stability issues. The model’s optimization process remains smooth and efficient, and convergence is not compromised.

#### 4.3.2. Effect of the Reduction Ratio *r*

**Figure 6:**
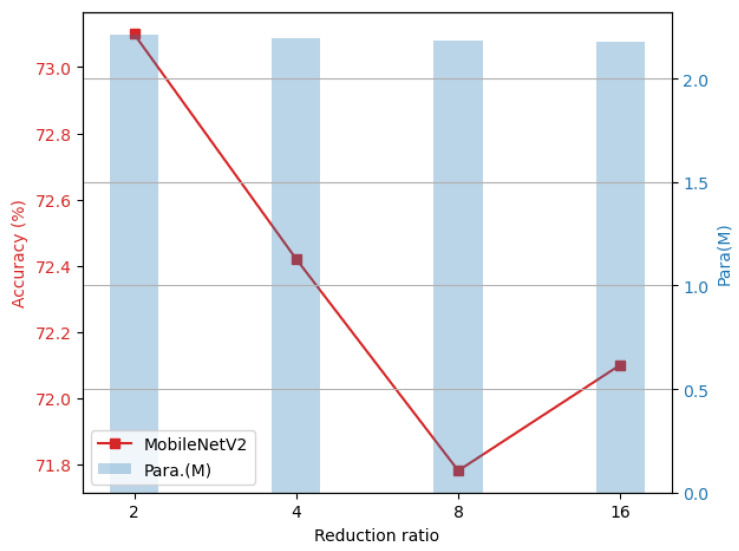
Relationship between trainable parameters and accuracy under different reduction ratios based on MobileNetV2 and CIFAR-10-C dataset.

In lightweight CNNs based on the GNTF, the reduction ratio *r* is a key structural hyperparameter that directly balances the reduction in trainable parameters with the preservation of model expressivity. Specifically, *r* is defined as the ratio of the total number of filters in the convolutional layer to the number of seed filters. For example, r=2 indicates that the seed filter accounts for half of the total number of filters, and the remaining filters are generated by performing nonlinear transformations on these seed filters. As shown in Figure 6, as *r* increases (i.e., the proportion of the seed filter decreases), the number of parameters that can be trained independently in the model decreases accordingly. However, when *r* is too large, the model’s representational capacity is severely limited, reducing robustness.

**Figure 7:**
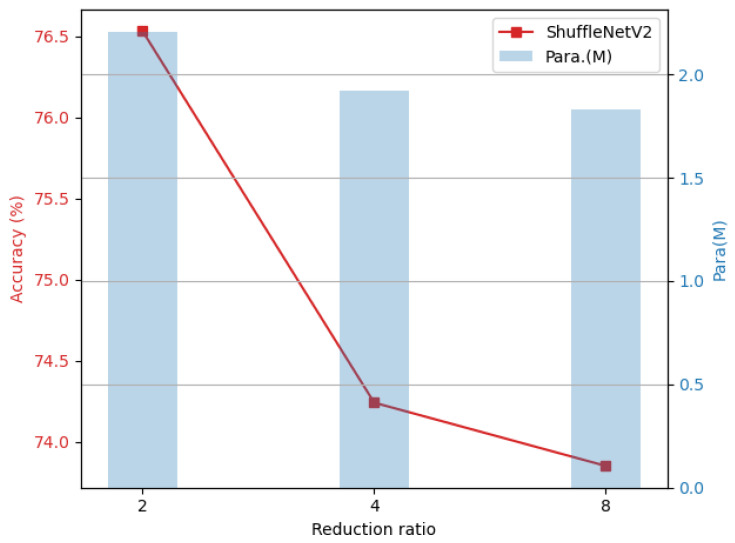
Relationship between trainable parameters and accuracy under different reduction ratios based on ShuffleNetV2 and CIFAR-10-C dataset.

This phenomenon has also been verified in classic lightweight models such as ShuffleNetV2 (as shown in Figure 7). In the ShuffleNetV2 model, some deep convolutional layers have 24 output channels, and 24 is not divisible by 16, so r=16 cannot be applied uniformly across these layers. To ensure that all layers use a consistent reduction rate, we set it to at most r=8 in our ShuffleNetV2 experiments. As *r* increases, the model’s number of trainable parameters decreases, but its robustness accuracy also decreases gradually. Therefore, when designing lightweight CNNs based on the GNTF, one should not simply pursue an extreme reduction in the number of parameters, but rather carefully balance the trade-off between compression ratio and model robustness. Based on this, the reduction rate was uniformly set to r=2 in the comparative experiment of this paper to achieve a good trade-off between parameter efficiency and robustness.

#### 4.3.3. Effect of β

**Figure 8:**
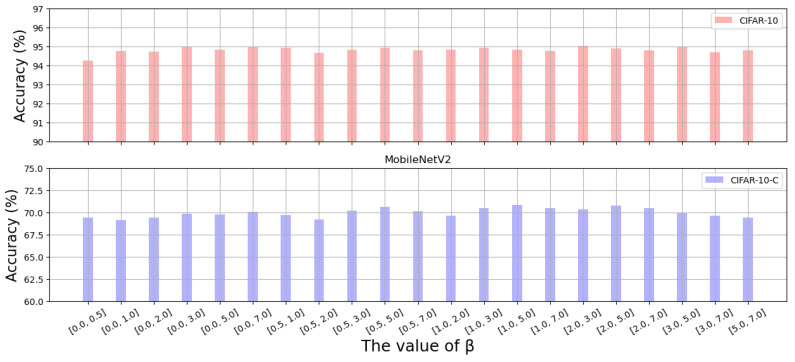
Impact of the range of the Monomial exponent. The Monomial exponent β is uniformly sampled from [a, b], where a and b are the lower and upper bounds.

**Figure 9:**
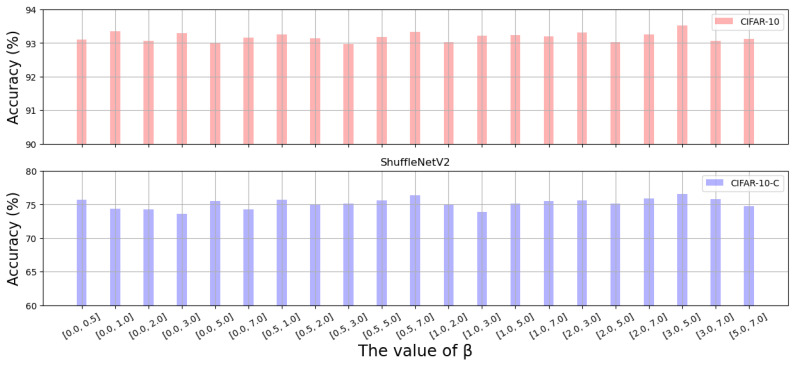
Impact of the range of the Gaussian exponent. The Gaussian exponent β is uniformly sampled from [a, b], where a and b are the lower and upper bounds.

In the experiment, the hyperparameters of the nonlinear transformation functions are selected via random sampling from the interval [a,b], where *a* and *b* denote the lower and upper limits of the parameter, respectively. We first evaluate the impact of the parameter setting on MobileNetV2. Experimental results for the hyperparameters of the Monomial function used by MobileNetV2 (Figure 8) show that the model achieves the highest accuracy on the CIFAR-10-C dataset when a=1 and b=5. In addition, we conduct similar experiments on the Gaussian nonlinear transformation function based on ShuffleNetV2. The results (Figure 9) show that the model achieves the best performance on the CIFAR-10-C dataset when a=3 and b=5. Based on the above results, we uniformly set a=1 and b=5 for MobileNetV2 experiments and a=3 and b=5 for ShuffleNetV2 experiments.

Furthermore, by comparing the model’s performance on CIFAR-10 (clean data) and CIFAR-10-C (corrupted data) across different parameter settings, we observed a consistent trend: the model’s robust accuracy on CIFAR-10-C is positively correlated with its standard accuracy on CIFAR-10. This demonstrates that our proposed GNTF does not impair the model’s ability to recognize original, clear data. On the contrary, it enhances feature stability and improves the model’s overall performance on both clean and corrupted data.

## 5. Conclusions and Future Work

This paper addresses the critical issue of lightweight convolutional neural networks’ susceptibility to environmental degradation in IoT and edge computing scenarios and proposes a robustness enhancement method based on a generative nonlinear transform filter (GNTF). This method breaks from the traditional approach to convolutional layers, relying entirely on data-driven learning of filter parameters. It designs a structured parameter-generation mechanism: only a subset of the network’s filters is used as learnable seeds, and the remaining filters are generated by applying a nonlinear transformation with fixed hyperparameters to the seeds. This embeds a prior structure, independent of the data distribution, into the model, thereby improving its generalization to external disturbances, such as noise and weather changes. This paper further clarifies two key prerequisites for the efficient application of this method: first, the generation mechanism should be applied primarily to the 3×3 deepwise convolutional layer in the lightweight model, which performs the core spatial feature extraction. Constraining this layer yields the largest robustness gain, whereas the impact on the 1×1 pointwise convolutional layer is limited. Secondly, the mathematical properties of the nonlinear transformation function itself are crucial, especially when the function passes through the origin (or can be translated to do so), which can improve performance. Experiments on multiple robust benchmark datasets, such as CIFAR-10-C, CIFAR-100-C, and ICONS-50, demonstrate that this method improves model accuracy on damaged data while reducing the number of trainable parameters in mainstream lightweight architectures, such as MobileNetV2 and ShuffleNetV2, thereby achieving a balance between reduced burden and increased efficiency.

However, this study has the following shortcomings: (i) the validity verification of the nonlinear transformation function is mainly based on experimental observation and lacks systematic theoretical support; (ii) the types of functions examined are limited, and the coverage is insufficient; (iii) the function adaptability analysis for specific tasks is not yet sufficient. In the future, we will: (i) expand the types of nonlinear transformation functions, including cases such as symmetric but non-zero center and asymmetric but zero center, and theoretically verify the effectiveness of zero-crossing and symmetric functions, and (ii) deeply analyze the applicability of different function characteristics to specific tasks, so as to establish a more complete task-oriented selection mechanism.

## Figures and Tables

**Table 1:** Effect of different configurations of GNTF.

Models	First-PW	Depth–Wise	Second-PW	Trainable Para. (M)	CIFAR–10	CIFAR–10–C
GNTF-MobileNetV2	✓			1.66	$94.64_{\pm 0.11}$	$72.41_{\pm 0.26}$
		✓		2.00	$94.76_{\pm 0.04}$	$73.10_{\pm 0.28}$
			✓	1.55	$94.27_{\pm 0.24}$	$72.02_{\pm 1.10}$
	✓	✓		1.62	$94.25_{\pm 0.01}$	$71.17_{\pm 0.01}$
	✓		✓	1.17	$94.15_{\pm 0.16}$	$71.34_{\pm 1.60}$
		✓	✓	1.52	$94.11_{\pm 0.13}$	$71.72_{\pm 0.15}$
	✓	✓	✓	1.14	$93.69_{\pm 0.35}$	$70.79_{\pm 0.48}$
GNTF-ShuffleNetV2	✓			1.74	$93.30_{\pm 0.82}$	$73.67_{\pm 2.15}$
		✓		2.20	$93.40_{\pm 0.76}$	$74.82_{\pm 0.23}$
			✓	1.66	$93.20_{\pm 0.59}$	$73.21_{\pm 2.42}$
	✓	✓		1.73	$93.50_{\pm 1.02}$	$73.02_{\pm 2.43}$
	✓		✓	1.28	$92.80_{\pm 0.96}$	$72.57_{\pm 1.00}$
		✓	✓	1.65	$92.80_{\pm 0.98}$	$72.79_{\pm 0.14}$
	✓	✓	✓	1.27	$92.80_{\pm 1.05}$	$71.01_{\pm 1.37}$

**Table 2:** Performance of different NTFs by simply shifting.

		GNTF–MobileNetV2	GNTF–ShuffleNetV2	GNTF–EfficientNet	GNTF–GhostNet
CIFAR-10-C	Mono	$72.02_{\pm 1.10}$	$74.82_{\pm 0.23}$	$72.92_{\pm 0.75}$	$73.32_{\pm 0.90}$
	Sine	$70.79_{\pm 2.40}$	$76.15_{\pm 0.81}$	$70.47_{\pm 0.19}$	$73.47_{\pm 0.18}$
	Sine–modify	$71.63_{\pm 1.36}$	$76.44_{\pm 0.47}$	$71.97_{\pm 0.16}$	$73.69_{\pm 0.01}$
	Sigmoid	$70.42_{\pm 0.94}$	$75.01_{\pm 1.50}$	$67.50_{\pm 0.33}$	$73.30_{\pm 0.42}$
	Sigmoid–modify	$71.57_{\pm 1.36}$	$76.24_{\pm 0.83}$	$72.19_{\pm 0.37}$	$73.45_{\pm 0.36}$
	Gaussian	$69.16_{\pm 2.64}$	$74.74_{\pm 0.62}$	$67.87_{\pm 2.13}$	$73.11_{\pm 0.37}$
	Gaussian–modify	$72.02_{\pm 1.68}$	$76.82_{\pm 0.41}$	$72.07_{\pm 0.59}$	$74.50_{\pm 0.24}$

The more effective results are displayed in bold.

**Table 3:** Comparison between standard and GNTF on CIFAR-10/-100-C, and Icons-50 datasets.

Models	Methods	Para. (M)	Accuracy (%)		
			CIFAR-100-C	ICONS-50	CIFAR-10-C
MobileNetV2	Standard	2.29	46.89	93.16	71.16
	GNTF	2.00	**47.08**	**93.22**	**73.30**
ShuffleNetV2	Standard	2.49	46.99	92.39	73.19
	GNTF	2.20	**47.94**	**92.62**	**76.53**
EfficientNet	Standard	3.60	45.77	91.07	70.26
	GNTF	3.28	**46.18**	**91.20**	**73.45**
GhostNet	Standard	3.91	48.17	93.72	71.40
	GNTF	3.90	**48.63**	**93.88**	**73.95**

**Table 4:** Severity Levels Comparison in EfficientNet Structure (Top: EfficientNet; Down: GNTF-EfficientNet) on CIFAR-10-C.

	Brightness	Contrast	Defocus Blur	Elastic Transform	Fog	Frost	Gaussian Blur	Gaussian Noise	Glass Blur	Impulse Noise	Jpeg Compression	Motion Blur	Pixelate	Saturate	Shot Noise	Snow	Spatter	Speckle Noise	Zoom Blur	MEAN
MEAN	91.81	62.25	75.98	80.66	80.5	74.79	66.33	36.42	55.82	54.66	80.24	70.38	76.80	89.93	49.88	80.40	84.20	55.00	69.18	70.26
	91.01	**67.71**	**79.63**	**82.94**	**83.83**	**76.25**	**70.15**	**46.11**	**60.55**	**59.63**	79.84	**74.18**	75.80	89.01	**57.93**	80.30	83.83	**62.44**	**74.68**	**73.45**
severity 1	93.18	91.53	92.85	86.95	92.14	88.31	92.90	72.33	58.86	81.69	86.21	86.62	91.16	89.89	82.51	88.07	90.92	82.89	82.38	85.86
	92.75	**92.00**	92.61	**87.87**	**92.37**	87.71	92.60	**75.27**	**63.68**	**82.59**	85.95	**87.52**	90.72	**90.11**	82.45	87.58	90.77	82.76	**85.79**	**86.48**
severity 2	92.88	82.36	90.38	86.86	88.95	82.67	82.99	46.33	60.49	70.34	81.83	76.99	86.98	87.30	71.18	79.12	86.6	66.66	78.09	78.89
	92.26	**86.37**	**91.19**	**87.69**	**90.84**	82.46	**86.04**	**56.67**	**65.15**	**72.31**	81.04	**79.92**	86.12	87.13	**73.90**	**79.39**	85.88	**71.20**	**82.06**	**80.93**
severity 3	92.29	70.73	83.47	81.04	84.79	72.53	68.84	26.06	61.14	61.47	80.08	65.99	84.04	92.95	42.03	81.97	83.13	57.09	70.22	71.57
	91.52	**78.58**	**86.31**	**84.05**	**87.92**	**74.2**	**74.83**	**38.32**	**66.09**	**64.03**	79.62	**70.53**	82.58	92.11	**53.46**	81.41	81.66	**64.70**	**75.93**	**75.15**
severity 4	91.38	48.34	69.66	75.71	78.62	71.07	52.89	20.46	48.83	38.45	77.77	65.9	70.59	91.20	32.31	79.02	84.20	40.15	63.07	63.14
	90.55	**59.65**	**77.00**	**79.15**	**82.89**	**72.97**	**60.35**	**32.62**	**53.09**	**46.54**	77.47	**70.69**	68.45	89.81	**45.45**	**79.15**	**84.70**	**51.64**	**69.90**	**68.00**
severity 5	89.30	18.28	43.55	72.75	57.99	59.37	34.02	16.91	49.80	21.33	75.33	56.39	51.23	88.32	21.39	73.81	76.16	28.19	52.16	51.91
	87.97	**21.95**	**51.05**	**75.94**	**65.11**	**63.91**	**36.95**	**27.68**	**54.72**	**32.70**	75.14	**62.24**	51.15	85.91	**34.37**	**73.98**	**76.16**	**41.88**	**59.72**	**56.76**

**Table 5:** On-Device Evaluation.

Models	Memory (G)	MACs (M)	Inference Time (s)	Power (W)	Energy (W·S)
ShuffleNetV2	1.03	12171.61	2.889	4.712	13.61297
GNTF–ShuffleNetV2	1.04	12171.61	2.889	4.686	13.53317

## Data Availability

The data used in this study are publicly available.
